# Patient-derived glioblastoma cultures as a tool for small-molecule drug discovery

**DOI:** 10.18632/oncotarget.27457

**Published:** 2020-01-28

**Authors:** Ling F. Ye, Eduard Reznik, Joshua M. Korn, Fallon Lin, Guizhi Yang, Kimberly Malesky, Hui Gao, Alice Loo, Raymond Pagliarini, Tom Mikkelsen, Donald C. Lo, Ana C. deCarvalho, Brent R. Stockwell

**Affiliations:** ^1^ Department of Biological Sciences, Columbia University, New York, NY 10027, USA; ^2^ Novartis Institutes for BioMedical Research, Cambridge, MA 02139, USA; ^3^ Center for Drug Discovery and Department of Neurobiology, Duke University Medical Center, Durham, NC 27710, USA; ^4^ Departments of Neurosurgery, Henry Ford Hospital, Detroit, MI 48202, USA; ^5^ Department of Chemistry, Columbia University, New York, NY 10027, USA

**Keywords:** cell death, chemical biology, glioma, cancer

## Abstract

There is a compelling need for new therapeutic strategies for glioblastoma multiforme (GBM). Preclinical target and therapeutic discovery for GBMs is primarily conducted using cell lines grown in serum-containing media, such as U-87 MG, which do not reflect the gene expression profiles of tumors found in GBM patients. To address this lack of representative models, we sought to develop a panel of patient-derived GBM models and characterize their genomic features, using RNA sequencing (RNA-seq) and growth characteristics, both when grown as neurospheres in culture, and grown orthotopically as xenografts in mice. When we compared these with commonly used GBM cell lines in the Cancer Cell Line Encyclopedia (CCLE), we found these patient-derived models to have greater diversity in gene expression and to better correspond to GBMs directly sequenced from patient tumor samples. We also evaluated the potential of these models for targeted therapy, by using the genomic characterization to identify small molecules that inhibit the growth of distinct subsets of GBMs, paving the way for precision medicines for GBM.

## INTRODUCTION

Glioblastoma multiforme is the most common form of primary brain cancer in adults and is a deadly disease associated with extremely poor prognoses [1]. After diagnosis, patients have a median survival of 15 months with current standard-of-care therapies of surgery followed by chemotherapy and radiation [2]. Therefore, new therapeutic strategies, specifically targeted therapy, need to be explored for this disease. To that end, previous studies explored *in vitro* compound sensitivities in glioblastoma using cell lines cultured in the presence of serum, such as those from the Cancer Cell Line Encyclopedia (CCLE [3]. However, it has been shown that GBM cancer cell lines cultured with serum poorly represent the gene expression profile and physiology of GBM tumors in patients, and exhibit considerable divergence from the original tumors from which they were derived [4]. We have previously demonstrated that glioblastoma-patient-derived neurosphere cultures (serum-free) are able to preserve the parental tumor somatic mutations and copy number alterations, including extra-chromosomal oncogene amplification [5]. We have also demonstrated that these patient-derived neurospheres can be used to conduct high-throughput screens using small-molecules [6]. Here, we sought to extend the usefulness of these models by analyzing the gene expression profiles and mutation status of these patient-derived models using RNA sequencing. We have found through RNA sequencing of GBM neurospheres that we can predict sensitivity to small molecule inhibitors in some cases, paving the way for novel targeted therapies in GBM.

## RESULTS

### Isolation and *in vitro* growth of patient-derived glioblastoma samples

**Figure 1:**
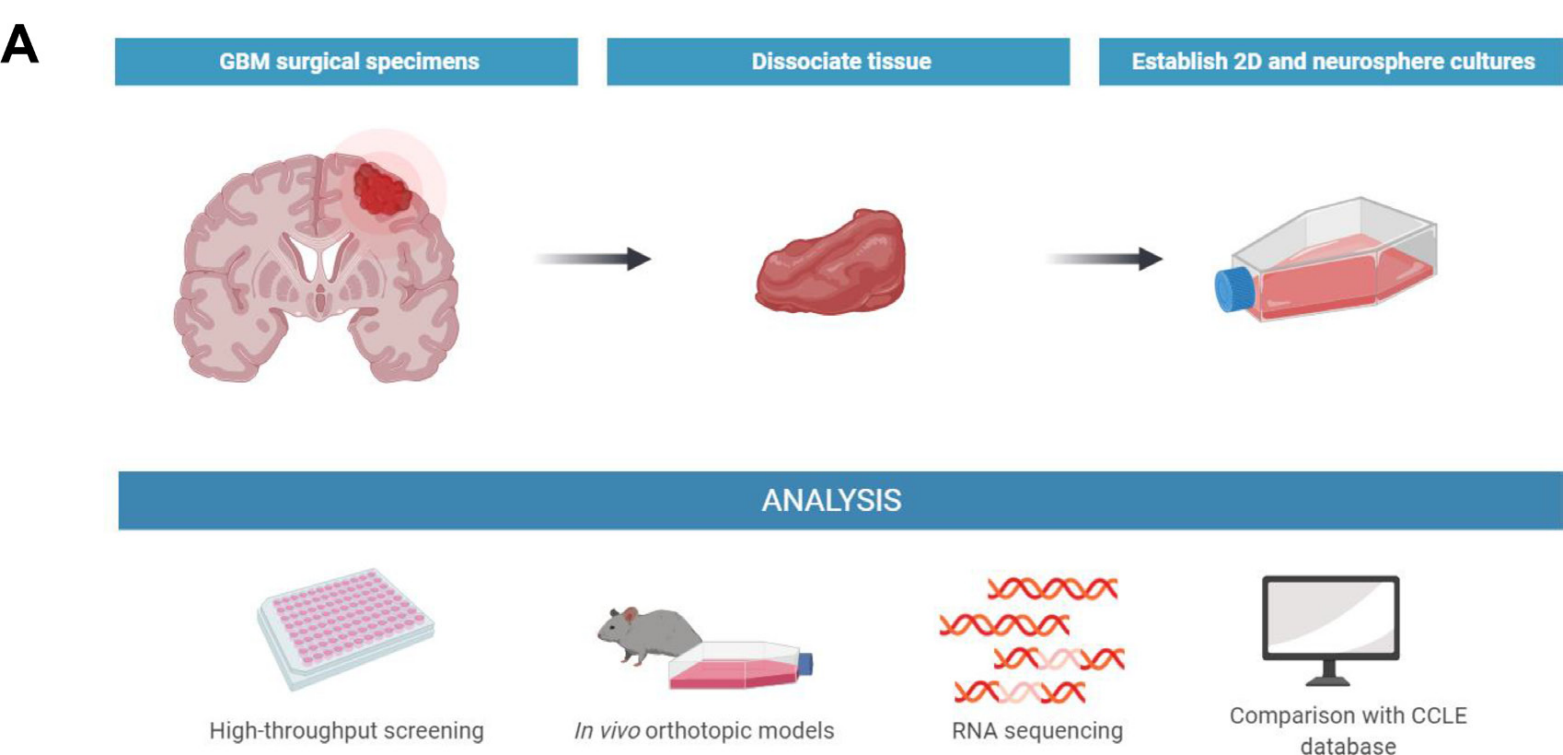
Isolation and growth characterization of new GBM models. (**A**) Illustration of the process of isolation, propagation, and analysis of patient-derived glioblastoma models.

Patient-derived glioma samples were collected at Henry Ford Hospital as previously described [7]. Dissociated cells from 22 glioblastomas and 1 oligodendroglioma were propagated, both *in vitro* and *in vivo*, to evaluate which would be amenable to *in vitro* high-throughput screening with small molecules (Figure 1A). If cells could not be consistently propagated as neurospheres in culture, laminin was added to the culture flask to a concentration of 1 μg/cm^2^, to allow cells to grow in 2D. We determined the optimal *in vitro* culture conditions as well as average doubling time, of each patient-derived model (Table 1). Of these, fifteen grew successfully as neurospheres in conditions suitable for high-throughput screening, three grew with the addition of laminin, and five were unable to survive long-term in culture under either condition. The doubling time of the cultures ranged widely between 84 hours (HF3026) and 625 hours for the oligodendroglioma model (HF3309).

**Table 1:** Overview of GBM growth characteristics *in vitro*

Cell line	Optimal growth condition for HTS	Average doubling time *in vitro* (h)
HF2303	Neurospheres	179
HF2381	Neurospheres	153
HF2414	None	N/A
HF2476	Laminin	122
HF2485	None	N/A
HF2561	None	N/A
HF2562	Neurospheres	165
HF2575	Neurospheres	87
HF2609	None	N/A
HF2790	Neurospheres	143
HF2876	Laminin	97
HF2885	Laminin	173
HF2906	Neurospheres	226
HF2941	Neurospheres	85
HF2998	Neurospheres	87
HF3013	Neurospheres	91
HF3019	Neurospheres	77
HF3026	Neurospheres	84
HF3037	Neurospheres	117
HF3177	Neurospheres	185
HF3216	None	N/A
HF3309	Neurospheres	625
HF3373	Neurospheres	134

Optimal growth conditions and average doubling time of patient-derived GBM cultures were recorded (*n* = 23).

### *In vivo* growth characteristics of orthotopic xenograft tumor models

**Figure 2:**
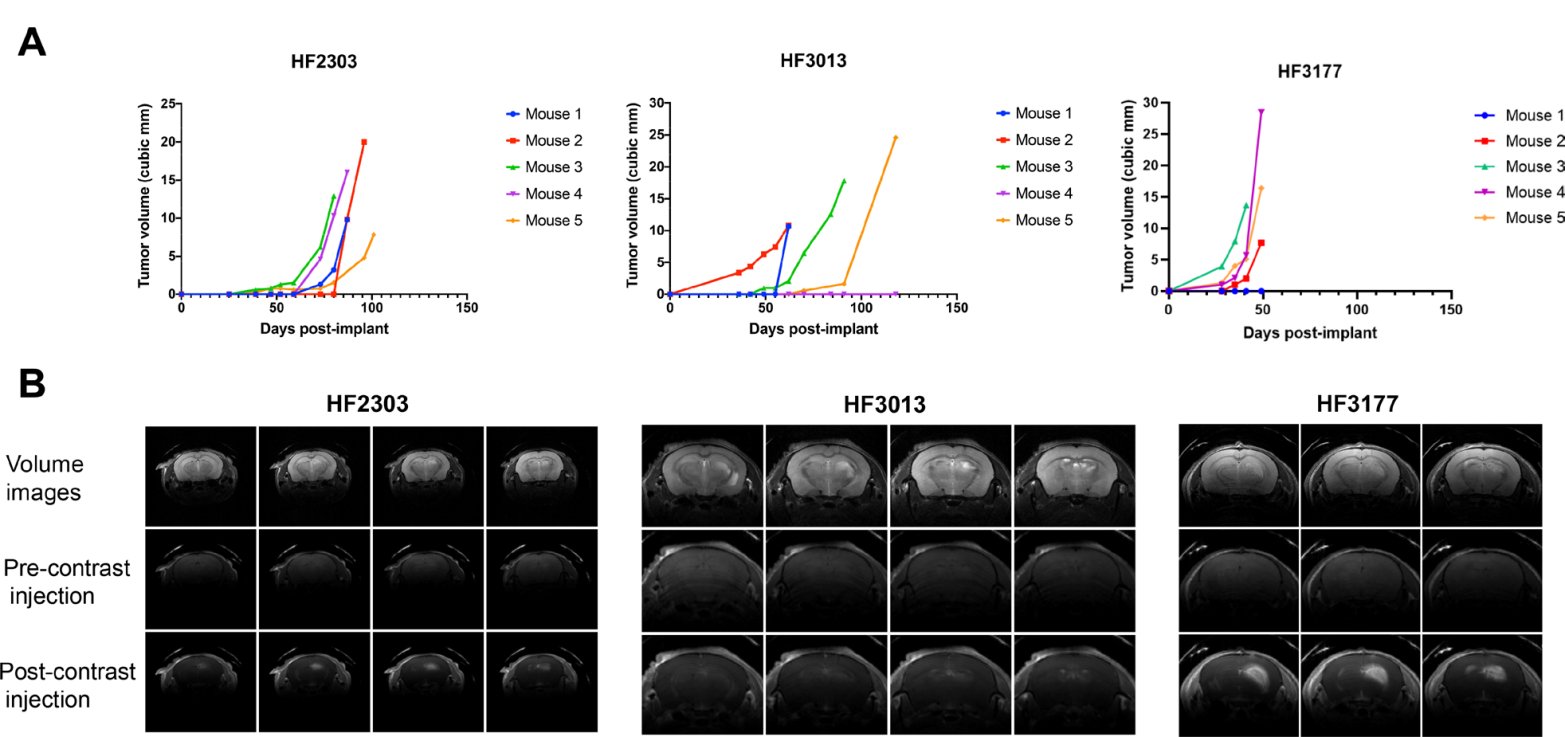
*In vivo* characteristics of orthotopic xenograft GBM models. (**A**) *In vivo* growth curves of orthotopic xenograft tumors grown in mice from patient-derived neurospheres HF2303, HF3013, and HF3177, with *n* = 5. (**B**) Representative MRI images, with and without gadolinium contrast, of orthotopic xenograft tumors grown in mice from cell lines HF2303, HF3013, and HF3177.

We then orthotopically implanted 15 of the GBM models into immunocompromised mice to evaluate which were able to form tumors *in vivo* and were suitable for further experimentation. We found that eight models were able to form at least one tumor visible by MRI when orthotopically xenografted into mice, within 28 days (HF3177) to 159 days (HF2876) post implant (Table 2). Following the injection of gadolinium contrast, we observed that tumors formed by 4 of the cell lines (HF2303, HF2609, HF3013, HF3177) appeared “leaky” on the images, indicating compromise of the blood-brain barrier within the tumors, a hallmark of GBMs [8]. The growth curves for three of the fastest-growing tumor models (HF2303, HF3013, HF3177) are shown in Figure 2A, and representative MRI images of these tumors are shown in Figure 2B.

**Table 2:** Growth characteristics of GBM cell lines after orthotopic xenograft in mice

Cell line	Number of mice with tumor	Number of days to first tumor formation	Contrast agent leakage seen on MRI
HF2303	5/5	47	yes
HF2381	0/4	N/A	N/A
HF2414	0/4	N/A	N/A
HF2476	0/5	N/A	N/A
HF2561	4/4	144	no
HF2609	2/4	80	yes
HF2790	3/5	40	no
HF2876	4/4	159	no
HF2885	0/3	N/A	N/A
HF2906	0/3	N/A	N/A
HF2998	1/4	42	no
HF3013	4/5	36	yes
HF3026	0/4	N/A	N/A
HF3037	0/5	N/A	N/A
HF3177	4/5	28	yes

For each cell line (*n* = 15), the tumor take rate in mice, the number of days to first tumor formation, and whether contrast agent leakage was observed on MRI were recorded.

### RNA-seq and mutational analysis highlights diversity of patient-derived glioblastoma samples compared to those from the CCLE

Next, we used RNA sequencing to analyze gene expression in each of the 23 patient-derived glioma models. In addition, tumors from four orthotopic xenograft models in mice (HF2303, HF2609, HF3013, HF3177) were sequenced, and three patient glioblastoma tissue samples taken directly from patients (HF2876, HF3177, and HF3216) were also sequenced. Additionally, raw RNAseq FASTQ files from 28 GBM cell lines from the CCLE were reprocessed using the same pipeline.

**Figure 3:**
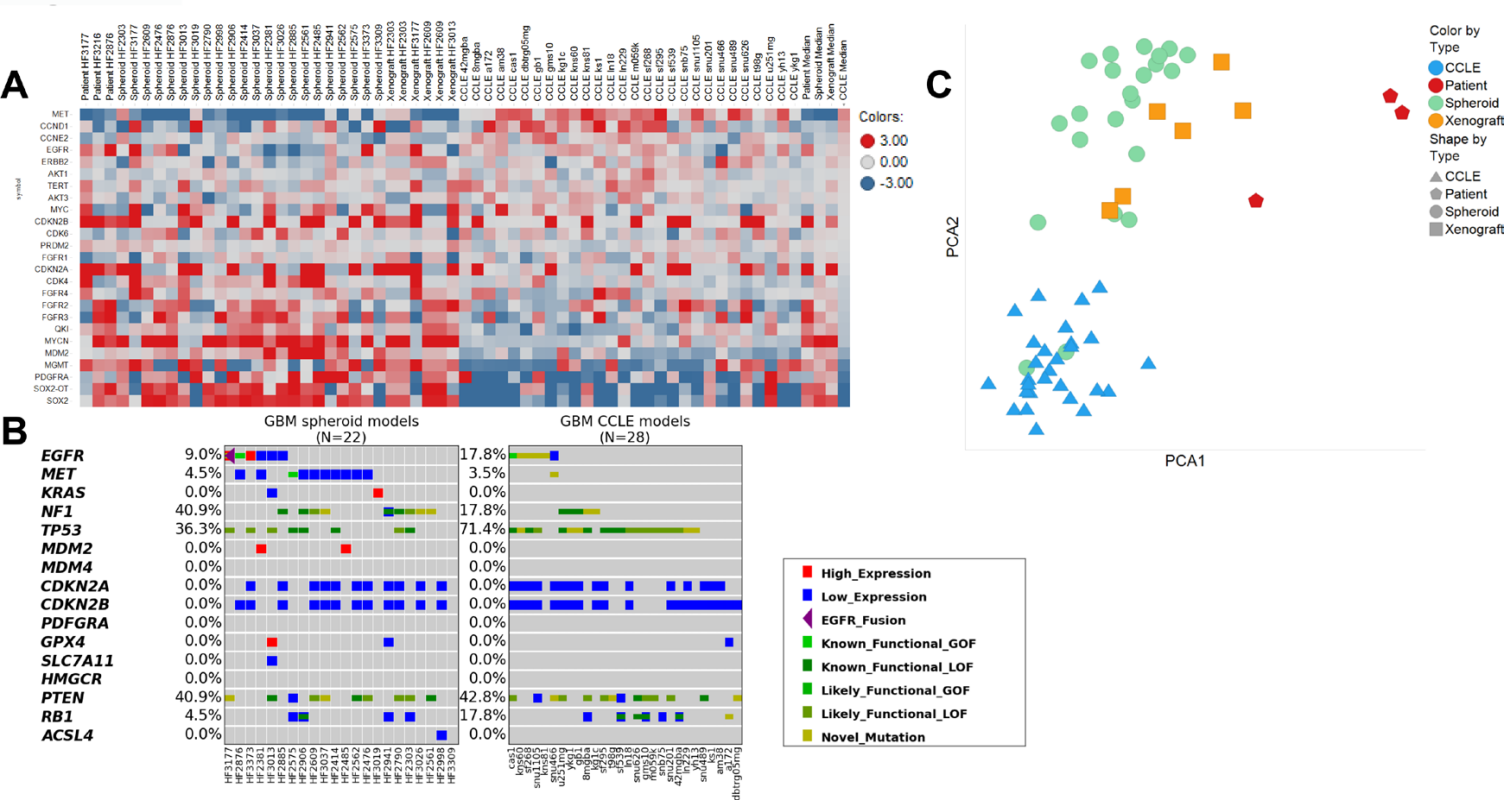
Comparison of gene expression and mutational status of patient-derived glioblastoma samples compared to those from CCLE. (**A**) Heatmap containing fold changes compared to the mean value of the expression of key genes of interest in patient-derived neurospheres vs. CCLE cell lines. Red signifies upregulation and blue signifies downregulation. (**B**) Mutational analysis of genes of interest in patient-derived neurospheres vs. CCLE cell lines. Dark green: loss-of-function SNV or indel. Lime: known gain-of-function SNV. Green: likely loss-of-function SNV (present in COSMIC). Mustard: SNV of unknown function. Purple triangle: EGFR fusion. Red: High expression (> 500 TPM or > 2IQR over median). Blue: Low expression (< 1 TPM or < 2IQR below median). (**C**) Principal component analyses of RNA sequencing data between GBM neurospheres sequenced as spheroid cultures (green), as orthotopic xenografts (orange), or as patient samples (red) and cell lines obtained from CCLE (blue).

We observed that the patient-derived models, as a whole, represented a more heterogeneous gene expression profile compared to the CCLE models, which better reflect the disease’s diversity. Analyzing genes relevant to GBM, we found high levels of MET mRNA expression in 21/28 CCLE lines, and in 2/23 patient-derived models (Figure 3A). MET gene is amplified in about 4% of GBMs, and mRNA overexpression is observed in about 11% [9]. This demonstrates that GBM cell lines grown in culture containing serum tend to become more mesenchymal while deviating from pro-neural tumors transcriptionally similar to oligodendrocyte progenitor cells, while also losing their stem cell-like qualities. This is consistent with the finding that pro-neural glioma stem-like cells found in GBMs, when cultured under conditions containing serum, showed an induction of mesenchymal gene expression signatures [10]. This effect was not observed in the spheroid model cultures, suggesting that serum-free culture of spheroid models can better preserve the genomic characteristics of GBMs in patients. Next, we examined the mutation status of several genes relevant to GBM and ferroptosis, because several ferroptosis-inducing compounds were included in the small molecule sensitivity screen (Figure 3B). Neurosphere cultured models were more likely to be *NF1* mutant, and less likely to be *TP53* mutant, compared to CCLE. We were also able to culture one model with an *EGFR* fusion and one with a gain of function *MET* mutation, neither of which exists in the CCLE GBM cohort. For genes related to ferroptosis, only three neurospheres expressed significantly high or low levels of *GPX4*, *SLC7A11*, or *ACSL4*, compared to one in the CCLE cohort. Finally, principle component analyses (PCA) showed that patient-derived neurosphere cultures, and even more so mouse xenografts, are transcriptionally more similar to patient samples than GBM cell lines in the CCLE (Figure 3C).

### MDM2 copy number, p53 mutation status, and EGFR mutation status predict cell line sensitivity to small-molecule inhibitors

**Figure 4:**
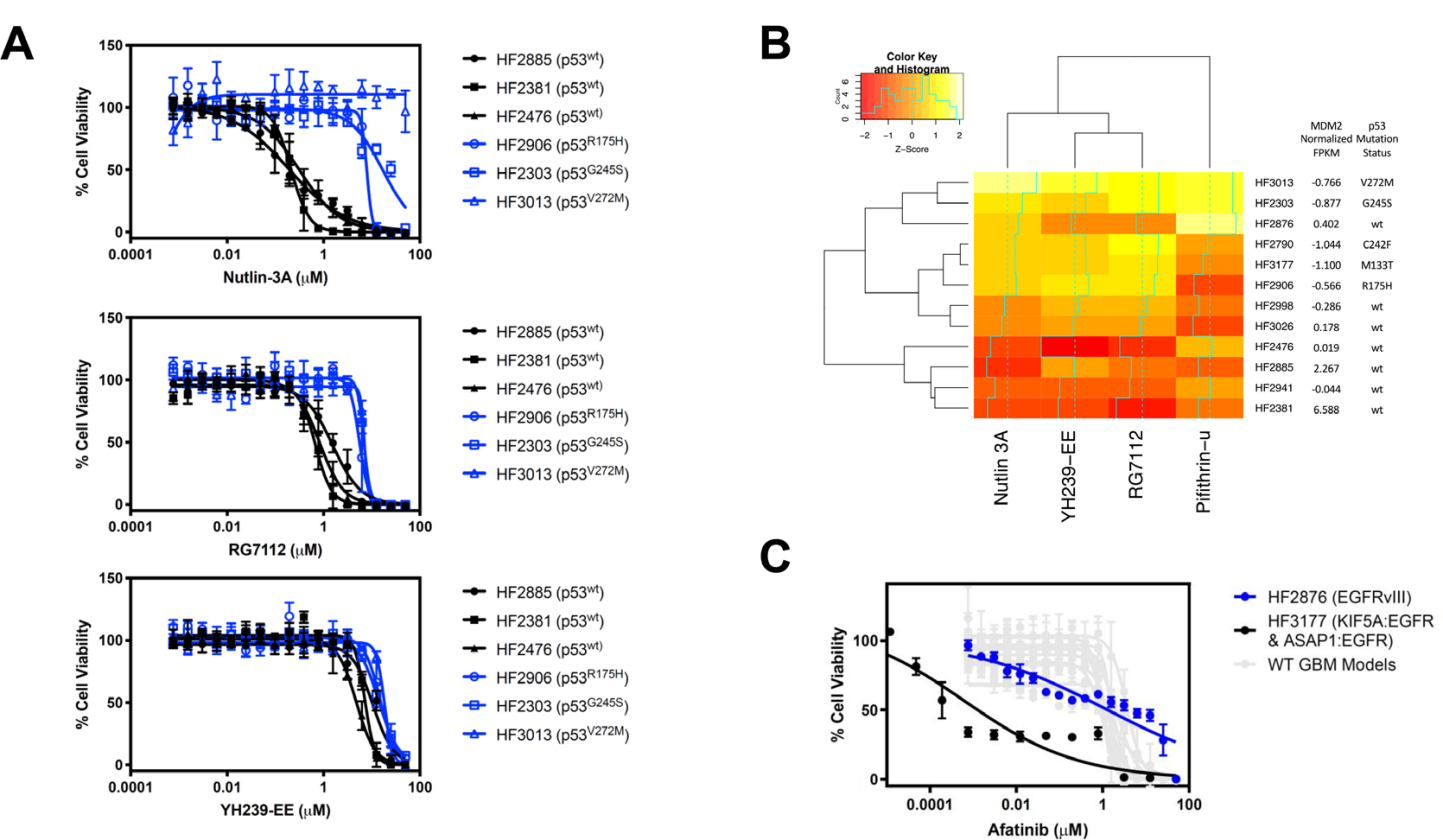
Discovery of MDM2 inhibitor sensitivity correlation with MDM2 copy number and p53 mutational status. (**A**) Dose response of the 3 most sensitive and 3 most resistant GBM neurosphere models to Nutlin-3A, RG7112, and YH239-EE. Among the more sensitive cell lines with wild-type p53 (black), HF2885 and HF2381 are also predicted to be MDM2-amplified by RNA sequencing. Data are plotted as mean ± SD, with *n* = 3 side-by-side experimental replicates. (**B**) Heatmap showing correlation between MDM2 copy number and p53 mutation status and MDM2 inhibitor differential sensitivity across GBM lines. Color values are a Z score of log10 (EC_50_) for each compound in each line. Darker colors represent more sensitive cell lines to the compound. (**C**) Dose response of two GBMs with EGFR alterations to the EGFR inhibitor afatinib. HF2876 (blue) has EGFRvIII (EC_50_=1.324 μM), whereas HF3177 (black) has KIF5A: EGFR and ASAP1: EGFR fusions (EC_50_=0.0006 μM). GBM models with wild-type EGFR (HF2885, HF2381, HF2476, HF2941, HF3026, HF2998, HF2790, HF2906, HF2303, and HF3013) had EC_50_ above 0.96 μM, as indicated in Table 4. Data are plotted as mean ± SD, with *n* = 3 side-by-side experimental replicates.

In our small-molecule screen using patient-derived GBM neurospheres, one of the most striking features was the potency and patient-model-selectivity of the MDM2-p53 interaction inhibitor class of compounds. For example, nutlin-3A yielded a >500-fold change in EC_50_ between the most sensitive (HF2381, EC_50_ = 212 nM) and most resistant (HF3013, EC_50_ > 100 μM) lines (Figure 4A and Table 3). When we compared the sensitivity profile across 12 neurosphere models with their respective RNA-seq data, we found a strong correlation between the MDM2 overexpression and p53 mutation status inherent to each cell line, and their sensitivity to the small-molecule inhibitors YH239-EE, RG7112, nutlin-3A, which disrupt binding of p53 to MDM2, as well as pifithrin-μ, which targets mitochondrial p53. Using hierarchical clustering, we observed that the models most sensitive to these compounds were those with highest MDM2 expression, as well as those with wild-type p53 (Figure 4B). On the other hand, the least sensitive cells were those with lower MDM2 predicted copy number and those with p53 mutations. From the combination of sensitivity data for small molecules and RNA-seq data, we may be able to better predict GBM patients that can benefit from targeted therapeutics.

**Table 3:** Sensitivity of GBM models to p53-MDM2 inhibitors (EC_50_ in μM)

Compounds	Pifithrin-μ	RG7112	YH239-EE	Nutlin 3A	MDM2 predicted copy number	p53 mutation status
HF2885	0.8531	0.04785	2.234	0.189	7.76	wt
HF2381	0.857	0.009132	1.038	0.2116	55.63	wt
HF2476	1.466	0.01356	0.4542	0.313	normal	wt
HF2941	1.361	0.04077	1.12	0.5516	normal	wt
HF3026	0.6887	0.1334	2.153	1.284	normal	wt
HF2998	0.8735	0.2632	2.8	1.809	normal	wt
HF2790	1.225	0.9405	3.879	6.943	normal	C242F
HF3177	1.024	0.8706	3.961	6.968	normal	M133T
HF2906	0.7136	0.6126	4.61	7.93	normal	R175H
HF2876	3.784	0.0818	1.959	10.73	normal	wt
HF2303	3.353	0.9435	3.881	17.02	normal	G245S
HF3013	3.343	1.007	6.758	100	normal	V272M

The sensitivity of 12 different patient-derived GBMs in 3D neurosphere culture was determined by dose-response testing with each compound in triplicate. Mean EC_50_ values in micromolar are indicated in Table 3.

Furthermore, we noted that a specific EGFR fusion conferred sensitivity to the small molecule EGFR inhibitor afatinib (Figure 4C). In our models, although two GBMs were found to have with EGFR alterations, namely HF2876 (EGFRvIII) and HF3177 (KIF5A: EGFR and ASAP1: EGFR fusions), we found a large difference in sensitivity between the two, with the latter about 2000 times more sensitive than the former (EC_50_=0.0006 μM for HF3117 vs. 1.324 μM for HF2876). GBMs that did not harbor an EGFR alteration were also relatively insensitive to afatinib treatment, with EC_50_ values ranging between 0.96 and 3.63 μM (Table 4). In summary, a specific EGFR fusion provides another example of correlating RNA sequencing data with small molecule compound sensitivity.

**Table 4:** Sensitivity of GBM models to afatinib (EC_50_ in μM)

	Afatinib sensitivity
HF2885	0.9631
HF2381	1.024
HF2476	2.207
HF2941	1.781
HF3026	1.505
HF2998	1.589
HF2790	1.004
HF3177	0.0006084
HF2906	1.592
HF2876	1.324
HF2303	2.305
HF3013	3.633

The sensitivity of 12 different patient-derived GBMs was determined by dose-response testing with each compound in triplicate. Mean EC_50_ values in micromolar are indicated in Table 4.

## DISCUSSION

Here, we describe the isolation and characterization of a panel of 22 patient-derived GBM models, which together represent a valuable tool for study of this disease. These cells can be grown *in vitro* either as neurospheres or in 2D culture on laminin or low serum, but these different culture conditions do not affect model sensitivity to a panel of small-molecule inhibitors of cell viability [6]. Here, we evaluated the capacity of these models to grow orthotopically in mice and demonstrated that at least 8 models were implanted with relatively high rate of tumor formation. As such, we suggest that these represent valuable model for the screening and discovery of new small-molecule drugs for GBM.

We analyzed the transcriptomes of the models using RNA sequencing. Comparison of their gene expression with cell lines from the CCLE revealed a greater diversity of expressed genes in these new models and more similar to those found in TCGA clinical GBM studies. In particular, while the classic cell culture models tend to universally overexpress certain stem cell markers and markers of EMT, this widespread pattern was not found in the patient-derived neurospheres. Therefore, the patient-derived models appear to be a better representation of the genetic heterogeneity of the disease in patients. Indeed, principal component analysis of the cultures grown under different conditions show that the patient-derived models, when grown as neurospheres or as xenografts, have expression profiles closer to those found in patient samples than CCLE samples.

Using small molecule inhibitors of the p53-MDM2 interaction, we found that the differential sensitivity of the panel of GBM models to small molecules could be correlated with MDM2 gene expression as well as TP53 mutational status. In this case, samples with higher expression of MDM2 were more sensitive to MDM2 inhibitors. Similarly, the MDM2 inhibitors were more potent in models with wild-type p53, as degradation of mutant p53 is less dependent on its interaction with MDM2 [11]. To further test the idea that compound sensitivity can be predicted by gene expression, we tested the EGFR inhibitor afatinib in the models, which were all wild-type for EGFR with the exception of two GBMs, which harbored EGFR amplification and either the constitutively active EGFRvIII variant (HF2876), or KIF5A: EGFR and ASAP1: EGFR fusions (HF3177). We found that afatinib selectively killed the GBM cell line with EGFR fusions, with a potency almost 2000-fold higher than those observed in the other GBMs (Table 4). Taken together, we expect that the availability of these patient-derived GBM models with a high degree of genetic and transcriptional diversity will enable a better preclinical basis to find potential novel targeted therapies for glioblastoma. These models are readily available from the Hermelin Brain Tumor Center live biobank at Henry Ford Hospital. De-identified information about tumor type, location, treatment status and other patient information are recorded in Supplementary Tables 1 and 2.

## MATERIALS AND METHODS

### Contact for reagent and resource sharing

Further information and requests for resources and reagents should be directed to and will be fulfilled by the Lead Contact Brent R. Stockwell (bstockwell@columbia. edu). The GBM models are available from the Hermelin Brain Tumor Center live biobank at Henry Ford Hospital through a Material Transfer Agreement (adecarv1@hfhs.org).

### Cell culture

Isolation, propagation and characterization of GBM neurospheres were performed as previously described [7].

### *In vivo* orthotopic xenograft

Female 8–12 weeks old athymic nude mice from Charles River Lab (Crl: NU (NCr)-Foxn1^nu^, Massachusetts USA) were used as hosts for xenograft implantation. Mice were anesthetized by isoflurane inhalation, received an injection of Loxicom analgesics (5 mg/kg SC) pre-operation, and positioned in a stereotaxic frame for implantation (Stoelting model 51730, Illinois USA). Skin over the skull was scrubbed with betadine and 70% ethanol to maintain aseptic conditions. A midline skin incision and a small hole was drilled in the skull over the injection site. GBM cells were resuspended in sterile PBS, and 300,000 cells in 5 ul was slowly injected into the cortex (3.0 mm ML, -1.0 mm AP, 1.5 mm DV from bregma) using a mounted Hamilton syringe. Skin incision was closed with nylon suture, and animals allowed to recover with *ad libitum* access to food and water. Mice were monitored weekly for clinical assessment scoring and body weight. Monitoring of tumor growth by MRI started 3–4 weeks post implant and was performed weekly. Mice were sacrificed before tumor burden associated symptoms developed.

### MRI imaging and data analysis

Magnetic resonance imaging (MRI) was performed on a Bruker Biospec 7.0T/30 cm NMR system (Bruker Biospin Corp, Billerica, MA) equipped with BGA12 gradients using a mouse brain cryoprobe. Paravision 5.1 software was employed for data acquisition. Imaging setup was head first, prone position.

For MRI measurement, each mouse underwent gas anesthesia with 1–1.5% isoflurane in an oxygen fed nose cone. Paralube eye ointment was applied to the eyes to avoid drying during the scan. A bellows was placed under the body to measure respiration and a temperature probe was inserted rectally. Temperature was maintained at 35.5–36.5C using a warm water bed. Respiration and temperature were monitored using PC-Sam software from SA Instrument, New York. An 80 cm microrhenethane catheter with a 28G needle for insertion into the lateral tail vein and a 1cc syringe loaded with a gadolinium-based contrast agent (Magnevist, Bayer, USA) at the other end was placed prior to start of the imaging session.

T2-Weighted RARE 2D sequence with consecutive axial slices was used for volumetric imaging and tumor evaluation. Slices=26, Slice thickness=0.5 mm, TR=3500ms, TEeff=52ms, NEX=4, MTX=256 × 256, Total acquisition time=7.5 mins.

Regional contrast agent uptake was assessed with a T1 weighted MSME sequence obtained before and repeated for 10 mins after a 50 uL lateral tail vein injection of a gadolinium-based contrast agent. The MSME was obtained with the same slice position and FOV as the previous T2 scan is obtained using parameters Slices=26, Slice thickness=0.5mm, TR=500ms, TE=10.1ms, NEX=1, MTX=128 × 128, ave=2, Total acquisition time=2.1 mins.

Paravision 5.1 software was employed for data processing and regions of interest were drawn manually for quantification of tumor volume. Blood brain barrier integrity was assessed by comparison of signal intensity in the tumor region of the brain between pre and post contrast MSME images.

All procedures were conducted in accordance with a protocol approved by the Novartis Institutes of BioMedical Research Inc. Institutional Animal Care and Use Committee (IACUC).

### RNA sequencing and data analysis

Total RNA was extracted from cells using the Qiagen AllPrep DNA/RNA Isolation Kit (catalog number 80204). Total RNA was then quantified using the Agilent RNA 6000 Nano Kit (catalog number 5067-1511) on the Agilent 2100 BioAnalyzer. Two hundred nanograms of high purity RNA (RNA Integrity Number 7.0 or greater) was used as input to the Illumina TruSeq Stranded mRNA Library Prep Kit, High Throughput (catalog number RS-122-2103), and the sample libraries were generated per manufacturer’s specifications on the Hamilton STAR robotics platform. The PCR amplified RNA-Seq library products were then quantified using the Advanced Analytical Fragment Analyzer Standard Sensitivity NGS Fragment Analysis Kit (catalog number DNF-473). The samples were diluted to 10 nanomolar in Qiagen Elution Buffer (Qiagen material number 1014609), denatured, and loaded at a range of 2.5 to 4.0 picomolar on an Illumina cBOT using the HiSeq^®^ 4000 PE Cluster Kit (catalog number PE-410-1001). The RNA-Seq libraries were sequenced on a HiSeq^®^ 4000 at 75 base pair paired end with 8 base pair dual indexes using the HiSeq^®^ 4000 SBS Kit, 150 cycles (catalogue number FC-410-1002). The sequence intensity files were generated on instrument using the Illumina Real Time Analysis software. The resulting intensity files were demultiplexed with the bcl2fastq2 software and aligned to the human transcriptome using Salmon [12] v 0.8.2 and the gencode version 25 basic transcriptome. Command line options to salmon quant were ‘-q–libType IU–seqBias–gcBias –useVBOpt’.

## SUPPLEMENTARY MATERIALS



## Abbreviations

GBM: glioblastoma multiforme
RNA: ribonucleic acid
CCLE: Cancer Cell Line Encyclopedia
MRI: magnetic resonance imaging
PCA: principal component analysis
EMT: epithelial-mesenchymal transition
