# Supplementary material for: Patient-derived glioblastoma cultures as a tool for small-molecule drug discovery
Source: Oncotarget. 2020 Jan 28;11(4):443–51. doi: 10.18632/oncotarget.27457 (PMC6996910; doi:10.18632/oncotarget.27457)
Supplement: Supplementary file 1 [file oncotarget-11-443-s001.pdf]

Patient-derived glioblastoma cultures as a tool for small-molecule drug discovery

SUPPLEMENTARY MATERIALS

Supplementary Table 1: De-identified disease and treatment information of GBM cell lines

De-identifier	Diagnosis	Location	Treatment status @ urgery	Treatment after surgery	Additional information
HF2303	GBM	L parietal	untreated	TMZ/RT	gliosarcoma
HF2381	GBM	R temporal	untreated	TMZ/RT Novacea A4QN	
HF2414	GBM	L temporal	untreated	TMZ/RT/ bevacizumab CPT-11	
HF2476	GBM	R frontal	untreated	TMZ/RT/ erlotinib / bevacizumab	
HF2485	GBM	R temporal	untreated	TMZ/RT/ bevacizumab/ CPT-11	
HF2561	GBM	R parietal	RT/TMZ	Adnexus CT-322	
HF2562	GBM	L frontal	untreated	RT/TMZ -> bevacizumab	gliosarcoma
HF2575	GBM	R temporal	RT/TMZ	bevacizumab/ CPT-11	gliosarcoma
HF2609	GBM	L temporal parietal	RT/TMZ/ cilengitide ->bevacizumab/irinotecan	null	
HF2790	GBM	R temporal	untreated	RT	
HF2876	GBM recurrent	R occipital	RT/TMZ/ hydroxy-chloroquine	irinotecan, bevacizumab	
HF2885	GBM	Cerebellum	untreated	null	
HF2906	GBM	L frontal	untreated	RT/TMZ	
HF2941	GBM recurrent	L temporal	TMZ/RT	bevacizumab	
HF2998	GBM	R temporal	untreated	RT/TMZ	
HF3013	GBM	R temporal	untreated	TMZ/RT/ DCVax ->MetMab	
HF3019	GBM	L frontal	untreated	RT/TMZ	*variable cell culture conditions
HF3026	GBM	R temporal	untreated	RT/TMZ	
HF3037	GBM recurrent	R parietal	RT/TMZ/ bevacizumab	TMZ/CCNU	

HF3177	GBM recurrent	R temporal	TMZ/RT followed by DCvax	RT/ CCNU	gliosarcoma
HF3216	GBM	R temporal	untreated	RT/TMZ	
HF3309	Oligoden-droglioma	R frontal	untreated	TMZ	progressed to GBM, tumor positive for IDH1 R132H, 1p/19q mutation; *variable cell culture conditions
HF3373	GBM	R frontal	untreated	RT/TMZ, -> Novo TTF	gliosarcoma

For each GBM (*n* = 23), the diagnosis, tumor location, treatment plan and additional information were recorded. *HF3019 and HF3309 were grown as 3D neurospheres for high-throughput screening, but grown as a monolayer with laminin and serum for RNA sequencing.

Supplementary Table 2: De-identified patient information of GBM cell lines

De-identifier	Age @ diagnosis (years)	Gender	OS (days)	TTP1 (days)	MGMT
HF2303	62	M	317	88	U
HF2381	66	M	180	85	M
HF2414	66	F	1481	196	U
HF2476	39	F	717	351	M
HF2485	54	M	432	150	M
HF2561	43	M	659	261	U
HF2562	30	M	632	213	U
HF2575	32	M	188	588	n/a
HF2609	46	M	655	291	U
HF2790	79	F	138	88	M
HF2876	62	M	558	167	U
HF2885	45	F	45	30	U
HF2906	54	F	339	null	U
HF2941	56	M	1255	249	n/a
HF2998	67	F	131	39	U
HF3013	59	M	270	192	M
HF3019	70	M	203	null	M
HF3026	64	F	169	98	U
HF3037	51	F	557	171	U
HF3177	45	M	646	88	U
HF3216	76	M	94	null	U
HF3309	45	M	1143	434	M
HF3373	71	M	318	null	M

For each GBM (*n* = 23), the age, gender, survival data, and MGMT methylation status (U for unmethylated, M for methylated) were recorded.
